# Heatstroke‐like symptoms in a patient with coronavirus disease pneumonia

**DOI:** 10.1002/ccr3.3461

**Published:** 2020-11-05

**Authors:** Ryo Deguchi, Hikoaki Ohba, Taiki Haga, Atsushi Ujiro

**Affiliations:** ^1^ Intensive Care Department Osaka City General Hospital Osaka City Japan

**Keywords:** coronavirus, extracorporeal membrane oxygenation, fever, heatstroke

## Abstract

While there are no clear indications for body temperature control during viral infections such as COVID‐19, if heat stress caused by COVID‐19 leads to organ failure, then proactive body temperature regulation may be an effective treatment option.

## Graphical abstract

The patient's remittent fever changed to sustained fever on hospitalization day 9. * addition of continuous renal replacement therapy to the patient's treatment. ** addition of VA‐ECMO.

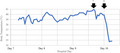

## 1 INTRODUCTION

Coronavirus disease (COVID‐19) is an emerging infectious disease that manifests with varying symptoms such as fever, fatigue, dry cough, and dyspnea.^1^ Reports have shown that fever is the most common symptom, appearing in approximately 90% of the reported cases. However, the natural history and progression of fever due to COVID‐19 have not been fully understood.^2^ Here, we present the case of a COVID‐19 patient exhibiting heatstroke‐like symptoms. There are no published reports demonstrating an association between COVID‐19 and heatstroke or supporting proactive body temperature regulation; hence, this case report may contribute to understanding COVID‐19 pathology and its treatment.

## 2 CASE HISTORY

A 47‐year‐old man presented with a 4‐day history of fever and dyspnea. Two days before hospitalization, he tested positive for severe acute respiratory syndrome coronavirus 2, assessed via reverse transcription polymerase chain reaction test. He also had a 5‐year history of untreated type 2 diabetes mellitus. On admission, his hemoglobin A1c level was 13%. He reported having no prior contact with other COVID‐19 patients.

**FIGURE 1:**
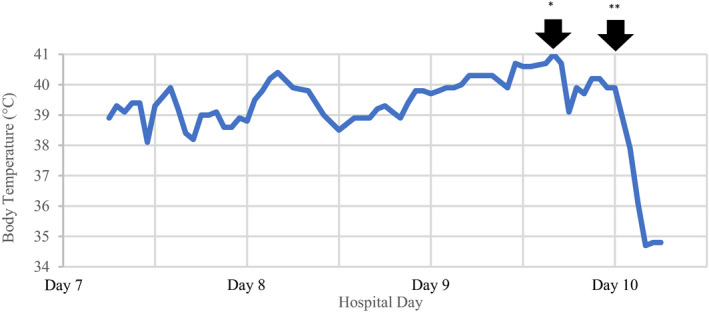
Patient's fever pattern after hospitalization. Although the patient had remittent fever on hospitalization days 6‐8, it changed to sustained fever on hospitalization day 9. *addition of continuous renal replacement therapy to the patient's treatment. **addition of VA‐ECMO. VA‐ECMO: venoarterial extracorporeal membrane oxygenation

The patient presented to the hospital with a body temperature of 38.3°C, heart rate of 90 beats/min, blood pressure of 132/83 mm Hg, respiratory rate of 24 breaths/min, and peripheral capillary oxygen saturation of 93% on ambient air. Chest computed tomography revealed ground‐glass opacities and bilateral patchy shadows consistent with those of COVID‐19 pneumonia. On the second day of hospitalization, the patient's respiratory condition worsened; hence, he was transferred to the intensive care unit (ICU) where oral intubation was initiated to provide artificial respiration. The patient had remittent fever, with a temperature ranging from 37.5°C to 40.4°C, on hospitalization days 6‐8. On day 9, he developed sustained fever, with a temperature of 40°C to 41°C (Figure 1), for which acetaminophen was ineffective. This was accompanied by a marked disturbance in consciousness. His systolic blood pressure dropped below 70 mm Hg, leading to warm shock. Respiratory condition further deteriorated on the night of day 9, with the partial pressure of arterial oxygen/fraction of inspired oxygen ratio decreasing from 202 mm Hg to 140 mm Hg. Large‐volume infusion of fluids was started along with the administration of vasopressors (noradrenaline, vasopressin, and adrenaline) and hydrocortisone. Venoarterial extracorporeal membrane oxygenation (VA‐ECMO) was initiated on the night of day 9 itself owing to worsening hemodynamics. Laboratory results (Table 1) revealed lactic acidosis, acute kidney injury, disseminated intravascular coagulation, hepatic dysfunction, and significant rhabdomyolysis. After the initiation of VA‐ECMO, the patient's sustained fever was rapidly alleviated, and the symptoms of multiorgan failure also began to improve gradually. VA‐ECMO support was withdrawn on day 22. Life‐prolonging treatment was withheld because of repeated severe cerebral infarction and hemorrhage that led to central nervous system damage. The patient died on day 30. Although favipiravir was administered to treat the patient's infection from the day 1 of his admission to the ICU, it was discontinued on day 7 because of the possibility of drug‐induced rhabdomyolysis. Broad‐spectrum antibiotics were administered from the day he was admitted to the ICU to prevent secondary bacterial infections. Sputum culture did not show the growth of any pathogenic organism.

**TABLE 1:** Clinical laboratory results of the patient

Variable	Reference range	Hospital day 8	Hospital day 9	Hospital day 10	Hospital day 11	Hospital day 12	Hospital day 13	Hospital day 15
Aspartate aminotransferase (U/L)	10‐40	54	76	1903	3399	2300	4249	1759
Alanine aminotransferase (U/L)	10‐49	24	27	205	446	328	679	387
Total bilirubin (mg/dL)	0.3‐1.2	1.1	1.1	3.4	6.7	6.0	6.9	11.8
Lactate dehydrogenase (U/L)	120‐246	456	539	3238	6990	2300	4249	1759
Creatine kinase (U/L)	56‐244	2227	3294	143 957	255 870	195 900	119 230	72 375
Creatinine (mg/dL)	0.7‐1.3	0.83	1.1	4.13	2.85	2.15	1.74	1.11
C‐reactive protein (mg/dL)	<0.3	25.7	22.1	10.06	10.21	4.73	2.03	2.92
Platelet counts (/µL)	172‐359	176	135	95	60	39	55	43
Prothrombin time international normalized ratio	0.85‐1.15	1.30	1.35	1.46	1.35	1.60	1.56	1.22
Activated partial thromboplastin time (sec)	24.0‐37.0	45.5	51.0	152.9^a^	170.0^a^	64.6^a^	71.6^a^	51.4^a^
Fibrinogen (mg/dL)	200‐400	526	471	268	258	144	118	168
Antithrombin Ⅲ (%)	80‐130	54	51	33	N/A	N/A	N/A	42
D‐dimer (µg/mL)	<1.0	17.9	17.5	34.7	15.2	5.1	3.5	6.5
Ferritin (ng/mL)	40‐165	3461	N/A	9504	N/A	5023	7340	1814
Lactic acid (mg/dL)	5‐14	20	14	94	53	78	53	23

^a^ Means using heparin.

## 3 DISCUSSION

We describe the case of a 47‐year‐old man positive for severe acute respiratory syndrome coronavirus 2 who presented with a 4‐day history of fever and dyspnea. His chest computed tomography findings were consistent with those of COVID‐19 pneumonia; laboratory test results and clinical findings revealed multiorgan failure and extremely elevated creatine kinase (CK) levels. The proportion of CK‐MB was not high; thus, we did not suggest heart diseases. However, several other differential diagnoses, such as malignant hyperthermia, malignant syndrome, and rhabdomyolysis, were thought of causing severe CK elevation. As the patient was not taking any volatile anesthetic (eg, halothane, isoflurane, sevoflurane, desflurane), succinylcholine, or antipsychotic drugs, malignant hyperthermia and malignant syndrome were ruled out. However, the CK elevation was later thought to be a symptom of rhabdomyolysis. Moreover, his initially remittent fever later developed into the sustained type, a sequela that has not yet been associated with previous COVID‐19 cases.

Furthermore, altered consciousness, multiorgan failure, and extreme hyperthermia with a temperature >40.5°C are symptoms consistent with heatstroke.^3^ In addition, the patient had a 5‐year history of untreated diabetes mellitus that could have had a role in the disease course and outcome of the patient, which should be investigated in subsequent similar reports.

In addition to these findings, the symptoms of multiorgan failure began to show signs of improvement upon regulation of the body temperature using VA‐ECMO. This suggests that the patient's symptoms may have been caused by heat stress. In the case of heatstroke, oxidative phosphorylation becomes uncoupled because of extreme hyperthermia and a variety of enzymes cease to function. A cytokine‐mediated systemic inflammatory response develops, and the production of heat‐shock proteins is increased. Blood is shunted from the splanchnic circulation to the skin and muscles, resulting in gastrointestinal ischemia and an increased permeability of the intestinal mucosa. Hepatocytes, vascular endothelium, and neural tissue are most sensitive to increased core temperatures, but all organs may ultimately be involved. Typical symptoms of heatstroke are a rectal core temperature of 105°F (40.6°C) or greater, multiorgan damage, and central nervous dysfunction,^4^ and in severe cases, circulation failure and disseminated intravascular coagulation are found.^5^ His clinical symptoms are consistent with heatstroke, but the absence of environmental factors that could have caused this condition makes proving an association between this case and heatstroke difficult. We thought that the heat dissipation mechanism could be disrupted by COVID‐19, which caused prolonged hyperthermia and acute physiological alterations such as circulatory failure, hypoxemia, and increased metabolic demands, and direct heat‐related cytotoxic effects could escalate, causing multiorgan failure. Despite all this, it must be noted that fever has beneficial effects, such as strengthening the immune response.^6^Proactive core body temperature regulation such as using extracorporeal apparatus is usually not conducted for hyperthermia but should be considered in cases of COVID‐19.

## 4 CONCLUSION

While there are no clear indications for body temperature control during viral infections such as COVID‐19, if heat stress caused by COVID‐19 leads to organ failure, then proactive body temperature regulation may be an effective treatment option. This report may help in understanding COVID‐19 pathology and treatment better owing to the possible demonstration of an association between COVID‐19 and heatstroke.

## CONFLICT OF INTEREST

None declared.

## AUTHOR CONTRIBUTIONS

RD: examined this case and drafted the manuscript. HO, TH and AU: reviewed and edited the manuscript.

## ETHICAL APPROVAL

The patient provided consent for the publication of this report.
